# NICE self-harm 2022 guideline: implications for ambulance staff

**DOI:** 10.29045/14784726.2023.9.8.2.57

**Published:** 2023-09-01

**Authors:** Faraz Mughal, Liam Clarke

**Affiliations:** Keele University; London Ambulance Service NHS Trust

After nearly 20 years, the National Institute for Health and Care Excellence (2022) has published its updated guideline for self-harm [NG225], which focuses on the assessment, management and prevention of repeat self-harm. Self-harm is closely associated with suicide, and is a significant international public health concern (Knipe et al., 2022). Self-harm is defined as self-injury or poisoning, irrespective of intent. The guideline utilised the latest evidence, synthesising the past two guidelines, and incorporating lived experience, to generate recommendations for health and social care, education, and criminal justice system settings. Ambulance staff may be the first professionals a person sees after self-harm, and therefore the initial assessment and management conducted can be crucial in preventing self-harm repetition. We highlight below the main recommendations for ambulance staff to consider.

An enduring principle is that patients are treated empathetically, with respect and compassion at every self-harm assessment. Each person deserves person-centred care regardless of how frequently they self-harm. Where immediate treatment of life- or limb-threatening physical injuries or illness are not needed, ambulance staff should consult the person’s care and safety plan, if applicable, and discuss with the person what help they may want (including assessment by their GP or mental health services). Ambulance staff should contact mental health specialists to obtain advice, and share relevant information with other professionals involved in the patient’s care, such as the means of self-harm, and the events preceding the self-harm episode.

When considering whether the patient should be conveyed to emergency hospital care, or if treatment from an alternative service is appropriate, ambulance staff need to consider any immediate safety concerns and assess if these can be appropriately managed in the community. Ambulance staff should not use risk assessment scores, scales or stratification to predict future self-harm or suicide, because of poor predictive utility, and instead, should undertake a focused assessment of individual clinical needs, and consider how and where best these are met. Ambulance staff should be vigilant for safeguarding concerns for the patient, as well as any dependants.

The guideline states that ambulance staff should have access to tailored self-harm training that includes topics such as communication, self-harm education, cultural sensitivity, and assessing the needs and safety of people. It has been shown that access to such training is poor, and therefore NHS ambulance trusts should prioritise the availability of training in the context of how common self-harm is (Rees et al., 2014). These recommendations may seem ambitious, or even unrealistic for ambulance staff, given the current pressures on services, but it is important to aim for excellent care in this group of patients who deserve better.
